# Supplementary material for: Effect of antiretroviral therapy on decreasing arterial stiffness, metabolic profile, vascular and systemic inflammatory cytokines in treatment-naïve HIV: A one-year prospective study
Source: PLoS One. 2023 Mar 17;18(3):e0282728. doi: 10.1371/journal.pone.0282728 (PMC10022802; doi:10.1371/journal.pone.0282728)
Supplement: S1 Table — (DOCX) [file pone.0282728.s001.docx]

|  | Included (n=20) | Not included (n=31] | P value |
| --- | --- | --- | --- |
| Age, years | 33 [26.2-45.5] | 31 [27-36] | 0.470 |
| Sex, male | 19 (95%) | 27 (87.1%) | 0.636 |
| BMI, kg/m^2^ | 25.3 ± 4.1 | 21.9 ± 3.3 | **0.002** |
| SBP, mmHg | 108 ± 14 | 112 ± 11 | **0.271** |
| DBP, mmHg | 66 [60-76.7] | 62 [60-68] | 0.119 |
| cfPWV, m/s | 7.3 [6.7-8.1] | 7.1 [6.6-7.9] | 0.434 |
| Smoking, n(%) | 14 (70%) | 20 (64.5%) | 0.685 |
| T CD4+, cells/mcL | 512 ± 324 | 472 ± 218 | 0.109 |
| T CD8+, cells/mcL | 977 [733-1374] | 1147 [688-1590] | 0.484 |
| CD4/CD8 | 0.39 [0.27-0.44] | 0.30 [0.23-0.44] | 0.088 |
| Viral load, Log10 | 4.6 ± 0.8 | 4.8 ± 0.6 | 0.243 |
| Months from diagnosis | 6.5 ± 3.3 | 6.6 ± 3.1 | 0.871 |
| TG, mg/dL | 167 ± 67 | 125 ± 47 | 0.018 |
| TC, mg/dL | 170 [124-200] | 143 [128-169] | 0.120 |
| c-HDL, mg/dL | 33± 9.4 | 34.1± 8.2 | 0.875 |
| c-LDL, mg/dL | 96.5 [70-105] | 84 [70-105] | 0.228 |
| hs-CRP, mg/dL | 2.8[0.60-6.6] | 2.6 [1.5-5.0] | 0.471 |

**S1 Table. Demographic and clinical characteristics of patients included and not included in the final analysis.**

Values are presented as median (IQR) and mean ± SD. BMI, body mass index; SBP, systolic blood pressure; DBP, diastolic blood pressure; cfPWV, carotid-femoral pulse wave velocity; TG, triglycerides; TC, total cholesterol; c-HDL, high-density lipoprotein cholesterol; c-LDL, low-density lipoprotein cholesterol; hs-CRP, high-sensitivity C-reactive protein.
